# The prenatal sex steroid theory of autism after 25 years

**DOI:** 10.1038/s41562-026-02437-0

**Published:** 2026-05-20

**Authors:** Simon Baron-Cohen, Alex Tsompanidis, Deepak P. Srivastava, Jonathan Mill, Madeline Lancaster, Dwaipayan Adhya, the APEX consortium, Deep Adhya, Omar Al-Rubaie, Carrie Allison, Bonnie Auyeung, Rosie Bamford, Simon Baron-Cohen, Richard Bethlehem, Tal Biron-Shental, Graham J Burton, Tereza Cindrova-Davies, Joanna Davis, Juan Pablo Del Rio, Lucia Dutan-Polit, Dori Floris, Alice Franklin, Lidia V Gabis, Daniel H Geschwind, Ramin Ali Marandi Ghoddousi, Jose Gonzalez-Martinez, Yeshaya David Greenberg, Genie Gu, Alexander EP Heazell, Rosie Holt, Matthew E Hurles, Yumnah T. Khan, Kyriaki Kosidou, Meng-Chuan Lai, Madeline A Lancaster, Mike Lombardo, Hilary C Martin, Jonathan Mill, Mahmoud Mousa, Roland Nagy, Kathy K Niakan, Niran Okewole, Adam Pavlinek, Marcin Radecki, David H Rowitch, Laura Sichlinger, Deepak P Srivastava, Alex Tsompanidis, Florina Uzefovsky, Varun Warrier, Elizabeth Weir, Yira Zhang, Varun Warrier

**Affiliations:** 1 Autism Research Centre, Department of Psychiatry, University of Cambridge; 2 MRC Centre for Neurodevelopmental Disorders, King’s College London; 3 Department of Basic and Clinical Neuroscience, Institute of Psychiatry Psychology and Neuroscience, King’s College London; 4 Department of Clinical & Biomedical Sciences, University of Exeter Medical School, University of Exeter; 5 MRC Laboratory for Molecular Biology, University of Cambridge; 6 Department of Psychology, University of Cambridge, Cambridge, UK

## Abstract

We first proposed the prenatal sex steroid theory of autism 25 years ago in order to account for a number of then-unexplained observations around autism, including (1) why autism is diagnosed more often in males than in females; and (2) apparent ‘male-type’ shifts in cognitive traits associated with autism, such as empathising and systemising. Here we review 25 years of research testing this theory. Early studies found that higher prenatal testosterone levels were associated with slower social, language and empathy development, greater attention to detail, stronger systemizing, and more autistic traits. Subsequent studies suggested that both prenatal androgens and estrogens are associated with autism. New methods in genetics and using stem-cell-derived neural organoids have further indicated the importance of sex steroid hormones for neurodevelopment, as well as atypical patterns in autism. These new findings support and open new lines of research into the prenatal sex steroid theory of autism.

Prenatal sex steroid hormones exert organisational effects on behaviour, brain development, structure and function. The earliest evidence for this came from animal models and *in vitro* studies, almost 50 years ago, in which testosterone was administered to rodents or their extant brain tissue and was reported to ‘masculinise’ aspects of behaviour and neuronal physiology^1,2^. Today, the effects of prenatal sex steroid can be modelled using human stem cell-derived neural organoids while administering different doses of sex steroids prenatally, to observe the effects of neuronal development^3^.

Sex steroids have different effects at different points of development^4^ and there is a gradual increase in their levels during the prenatal period. In a human fetus with a Y chromosome, which is usually (but not always) male, the developing testes start producing androgens. This leads to a surge in testosterone, between 8-20 weeks of gestation, which is associated with the development of male external genitalia and with the initial masculinization of the brain. This critical period is therefore referred to as the ‘masculinization programming window’ or MPW (Figure 1).

In parallel, estrogens are formed from androgens in the placenta and in the brain (via aromatisation) and their concentration increases gradually throughout pregnancy. Together with androgens, estrogens exert effects on neurons, synapses and glial cells via several different receptors^5^. Findings in human neural organoids^3^ indicate that androgens regulate the rates of neuronal proliferation and apoptosis *in vitro*, while estrogens affect the number of neuronal dendritic spines, neuronal migration and neurite outgrowth, as shown in multiple studies over the years^6,7^ (recently summarised in reviews by our team^8,9^).

While these models cannot fully capture the complexity of the prenatal environment, they are generally consistent with animal models^10^ which first defined the effects of sex steroid hormones in neurodevelopment and sex differentiation (Figure 2).

In this article we summarise the evidence gathered over a 25-year period showing associations between prenatal sex steroid hormone levels and autism-related outcomes in humans. We explore if these associations can partly explain why an autism diagnosis is more common in people assigned as male at birth (hereafter referred to as ‘boys’ or ‘men’ or ‘males’ although we recognise that many autistic people do not identify with their sex assigned at birth). Under-diagnosis of autism in people assigned as female at birth (hereafter referred to as ‘girls’ or ‘women’ or ‘females’, with the same caveat regarding gender identity as before) has historically partly reflected clinical and sociocultural factors, such as reduced awareness by clinicians of how autism manifests differently in females compared to males, and because of greater ‘masking’ or camouflaging of autistic traits in females, such that they are more likely to remain undiagnosed or misdiagnosed^11^.

However, despite the awareness of these features in girls and women now being greatly improved, the sex ratio in prevalence of autism persists. Over and above these clinical and sociocultural factors, we lay out the evidence that prenatal sex steroids levels alter neurodevelopment and predict both the number of autistic traits an individual has, and their higher likelihood of receiving an autism diagnosis. We highlight how the early findings that focused primarily on a role for androgens have been extended to the prenatal sex steroid theory of autism, to include prenatal estrogens as well. Since then, additional findings consistent with the theory have been published, as presented below, including from clinical studies, genetics, and experiments using model systems of the developing brain. The latter have only relatively recently become possible in human developmental neuroscience.

## Fetal testosterone regulates neurodevelopment in the general population

Twenty-five years ago, we set up the Cambridge Prenatal Testosterone Project, a longitudinal prospective cohort study, to test if prenatal testosterone levels are associated with later brain and behaviour outcomes in human children^4^. We measured prenatal testosterone in the amniotic fluid of pregnant women who were referred for amniocentesis by clinicians as part of routine prenatal care. We measured this hormone in amniotic fluid, as this was the most ethical way of measuring it in human fetal circulation during gestation.

Testosterone levels in amniotic fluid show marked sex differences^12^, since the male fetus produces testosterone prenatally from the testes, as well as their developing adrenal glands, whereas females only rely on the latter. Amniotic fluid is in osmotic equilibrium with fetal plasma in early pregnancy, as skin is not fully keratinised until approximately 25 weeks gestation^13^. Whilst women who are referred for amniocentesis are not a random population, often being older than 35 years old, we reasoned that this was the only ethical way to measure fetal sex steroid circulation directly, in a clinical setting, without additional risk of harm to the fetus. Given that amniocentesis is usually conducted between the first and early second trimester, it can capture the MPW, while minimising the variance related to gestational age of the fetus, or maternal age.

At follow-up we found that children with higher testosterone levels in amniotic fluid had reduced frequency of eye-contact at 12 months, reduced vocabulary at 18 and 24 months of age, poorer social relationships with other children at 4 years old and lower empathy scores at 8 years old. The direction of this statistical association was reversed for non-social traits: the higher the child’s amniotic testosterone, the narrower their interests, the greater their attention to detail, and the stronger their interests in inanimate, rules-based systems (systemizing)^14^. In addition, amniotic testosterone levels positively correlated with autistic traits both in toddlerhood (age 18-30 months) and in childhood (age 4 to 10 years old)^15^. Importantly, these statistical associations were significant in models that controlled for sex, and so are not confounded by baseline sex differences in testosterone.

Furthermore, we found associations between amniotic testosterone levels and the volume of specific brain regions^16^, such as the planum temporale (involved in language), the superior temporal sulcus (involved in ‘theory of mind’), and the amygdala (involved in emotion processing and empathy). Whilst it was already known that experimental manipulation of prenatal sex steroids, through castration of a male mouse or rat, or hormone administration to a female mouse or rat, changes brain development in the animal^1,17^ – experiments that would be unethical to conduct in humans – this was the first demonstration of an association between amniotic testosterone levels and brain development in humans. This effect of testosterone on neuronal proliferation is further supported in experiments of direct hormone administration in human stem-cell derived neural organoids, a model system of the developing brain ‘in a dish’^3^.

## Prenatal sex steroid levels are statistically associated with autism

**Figure 3:**
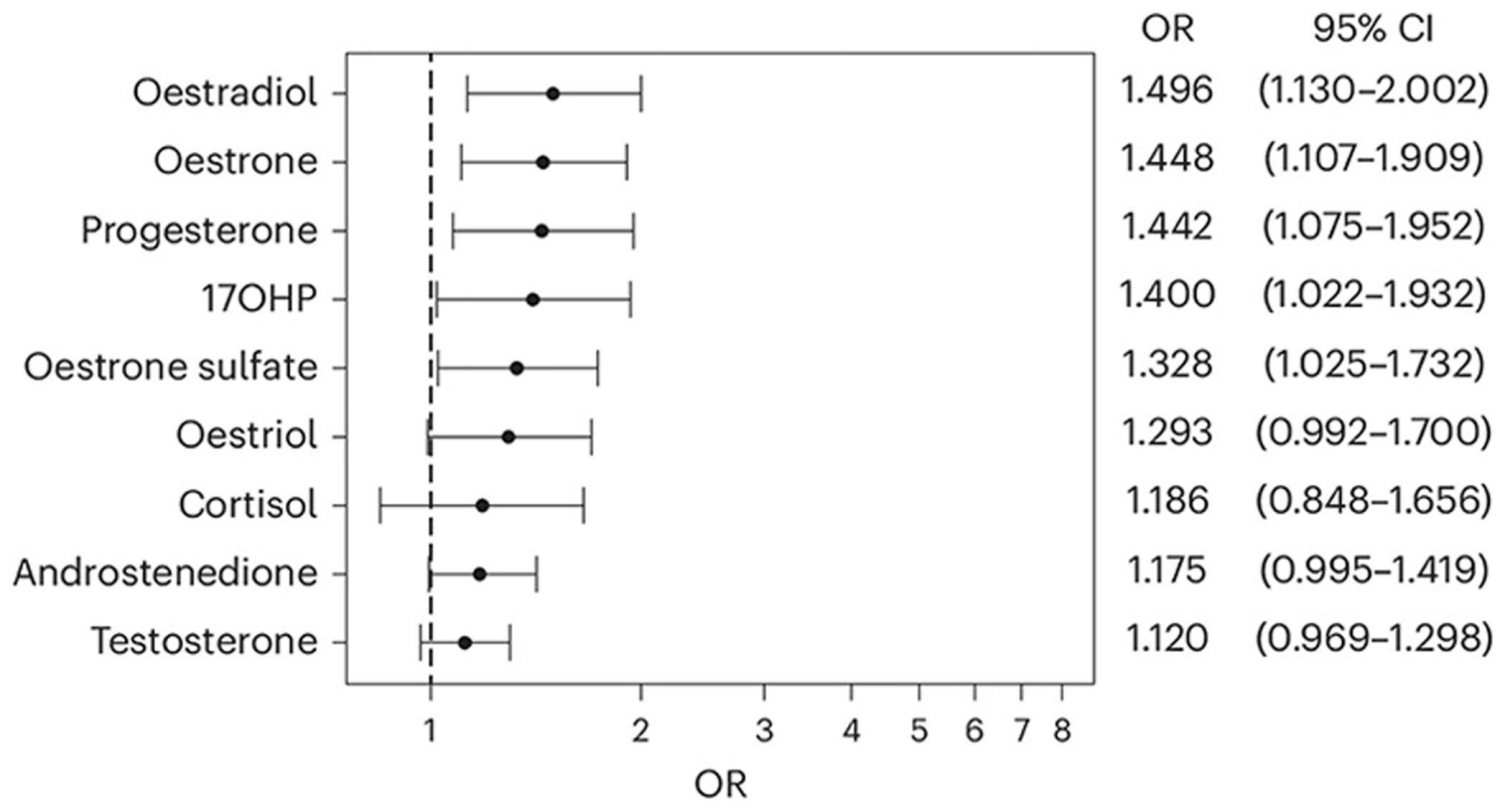
Amniotic steroid levels and autism. Odds ratios of prenatal sex steroids in autistic men, based on and reprinted from the case-control comparison in the Danish Biobank^19^.

To study prenatal sex steroids in cases of clinically diagnosed autism, in the Danish Biobank Prenatal Sex Steroids Study we retrospectively linked autistic males in Denmark to amniotic fluid samples that had been collected via amniocentesis over many decades, and which had been stored in the State Serum Institute in Copenhagen. This provided a sample size sufficient to test our prediction that there would be elevated prenatal sex steroid levels in autistic males. We compared the levels of several steroids in amniotic fluid that are part of the testosterone synthesis Δ4 pathway (progesterone, 17 alpha hydroxy progesterone, androstenedione, and testosterone). Their levels correlated with each other and, when examined together, were significantly elevated in pregnancies that resulted in an autism diagnosis^18^. We subsequently had the opportunity to extend this analysis to prenatal estrogens, since as mentioned earlier, these are rapidly synthesized from androgens via the action of the enzyme aromatase in the brain and placenta. We found that when examining each hormone separately, estrogen levels were more statistically predictive of autism likelihood than the androgens alone, but when examined together, they showed a large degree of interrelatedness, with latent factor analysis indicating elevated steroidogenesis across all pathways in autism^19^ (Figure 3).

This statistical association between prenatal estradiol and autism has also been replicated in an analysis of maternal samples from an independent longitudinal cohort in the US, which included pregnancies of autistic males^20^. Furthermore, in the Cambridge Ultrasound and Pregnancy (CUSP) Study^21^, we found an association between maternal serum estradiol levels during pregnancy and the autistic traits of their sons at 18 months. Finally, higher sex steroids have also been found in the early postnatal period, as shown for several androgen analytes (e.g., testosterone or androstenedione) in the meconium of boys with high autistic traits^22^.

It is important to note that some studies have not replicated these associations with traits or diagnostic likelihood. A small study found no relationship between testosterone levels in amniotic fluid and later autistic traits^23^. This may be due to limited sample size to detect an association, as well as the considerable variability of sex steroid levels, which may disproportionately impact studies that rely on a single measurement per participant. In addition, no relationship was found between sex steroids measured in umbilical cord blood after birth and the autistic traits of the children^24,25^. This again may reflect insufficient statistical power, or neonatal steroid levels being confounded by the onset of labour, a process that often involves reductions in or a functional withdrawal to the effects progesterone^26,27^. In addition, when examining sex steroids together, these studies did not separately test estrogens and androgens, opting instead to analyse the ratio between the two, rather than their additive effect. This may be important as the relationship between estrogen levels and autism may depend on the timing of pregnancy and the type of autism in question.

Furthermore, while our team and others have shown elevated prenatal estrogens, such as estradiol, both in amniotic fluid and in maternal plasma^19–21^, other studies of the maternal circulation during pregnancy have had more mixed findings. A study of clinical records in California found lower levels for maternal estriol and a U-shaped association for human choriogonadotropin (hCG)^28^, the placental hormone that regulates steroidogenesis in the first third of pregnancy. In addition, a study in Sweden found a similar result in earlier pregnancy (mean 10 weeks’ gestation), namely with lower maternal estradiol in cases of autism with intellectual disability^29^. Yet, the same study reported higher maternal 17-hydroxyprogesterone in cases of autism without intellectual disability, which correspond to 75% of the diagnosed population. Progesterone regulates steroidogenesis in the second and third trimester and is often positively associated with both androgens and estrogens^19^.

Taken together, the evidence is largely consistent with our initial report for an elevated ‘steroidogenic’ factor in autism that is comprised of several sex steroids downstream of progesterone^18^. This may be more apparent in males, in autism without intellectual disability, and particularly when steroids are measured in the early second trimester of pregnancy (after 14 weeks in human gestational age).

## The placenta as the source of elevated steroids

**Figure 4:**
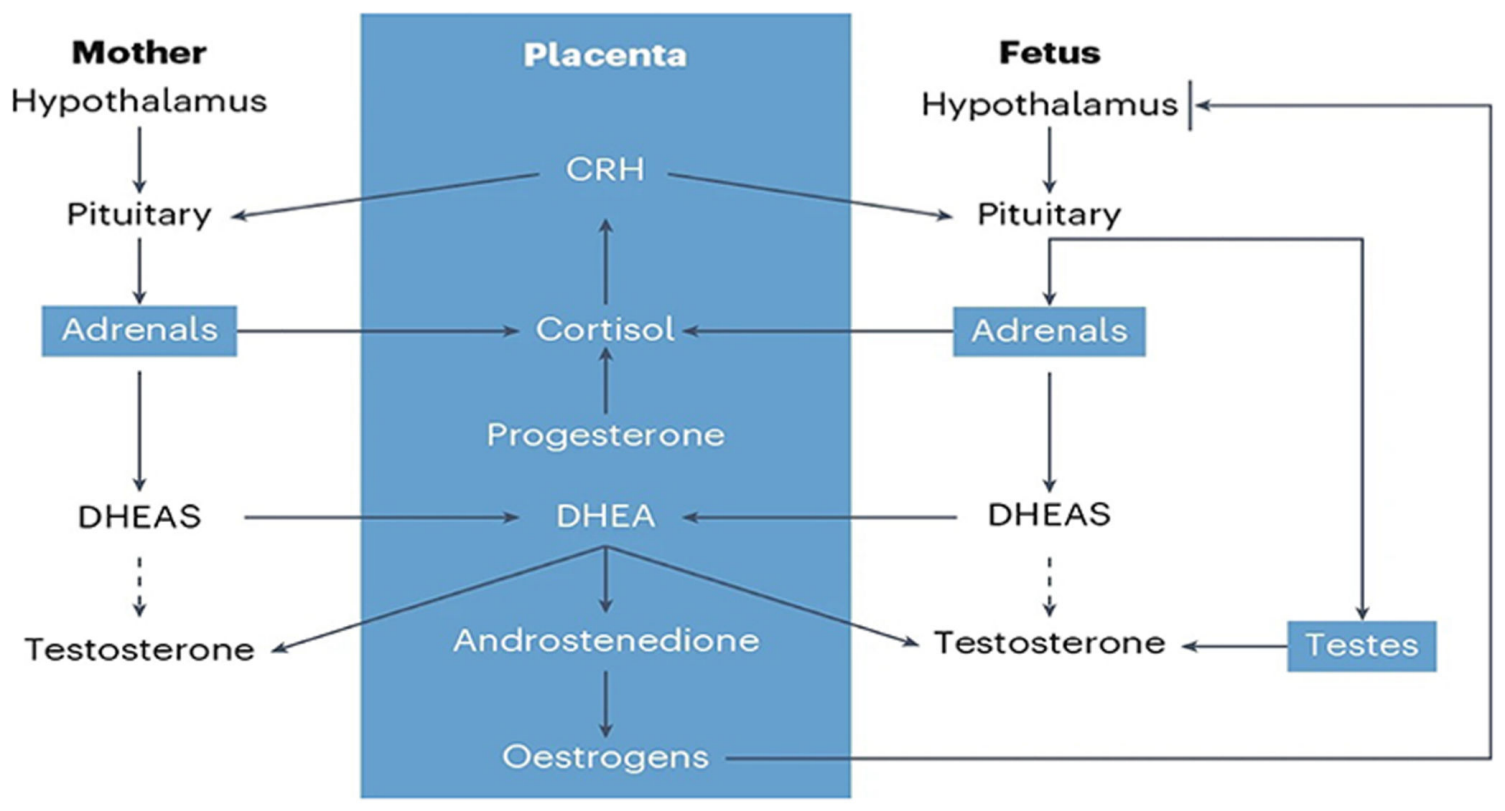
Prenatal steroidogenesis. The maternal, placental and fetal units interact via placental CRH (corticotropin-releasing hormone) and adrenal DHEA (dihydrotestosterone) to maintain the synthesis of prenatal sex steroids in the second and third trimesters. In pregnancies of boys, additional testosterone is introduced via gonadal production during the MPW.

The synthesis of sex steroids during gestation involves three different sources: the fetus, the mother, and the placenta (Figure 4). Steroid production gradually increases throughout pregnancy for both boys and girls, with the fetal and maternal adrenals producing steroid precursors that are converted into androgens and estrogens by the placenta. As mentioned above, in pregnancies of males, sex steroids are also produced by the testes, which synthesise high levels of testosterone from 8-20 weeks gestational age (during the MPW)^30^, which is then further converted to the more potent form of dihydrotestosterone (DHT). This affects physical development, contributing to sex differences in anatomical features such as the genitalia, overall body size and more specific anatomical features as the anogenital distance (AGD) of the pelvic floor^31,32^.

Steroids are also produced in and affect the brain. For example, studies of brain growth show that boys have larger brain volume at birth, independent of sex differences in birth weight^33^, while studies in human postmortem samples demonstrate that the brain can synthesise estrogens locally via the aromatisation of circulating androgens^34^. This conversion may be particularly relevant for mediating the effects of estrogens on neuronal migration^35^. In addition, the expression of aromatase in both glial cells^36,37^ and in cells of the choroid plexus^38,39^ may indicate complex interactions between different cell types in the brain (e.g., paracrine steroid metabolism) that warrant further research.

High levels of circulating estrogens may then be associated with other factors that increase prenatal steroidogenesis (e.g., maternal or placental health) or with insufficient negative feedback on the HPG axis (e.g., due to saturation of the estrogen receptor or partial insensitivity)^40^. Interestingly, this effect of estrogens on the hypothalamus may rely on GABA-ergic signalling^41^, which has also been implicated in clinical association studies of autism^42,43^. In addition, candidate gene studies have shown a post-mortem downregulation of the estrogen receptor in the brains of autistic individuals^44^, while estrogen treatment appears to ‘rescue’ gene pathways related to autism in two animal models^45,46^. Further research is needed to understand the relevance of estrogens in neurodevelopment, their precise mechanism and whether their potential dysregulation in autism is also associated with ‘male-type’ shifts in brain structure and cognition^47^.

Genetic factors in the mother and/or in the child are also relevant as they may increase the likelihood of endocrine conditions and contribute to variation in the levels and sensitivity to prenatal sex steroids. For example, common genetic variants associated with the regulation of complex endocrine traits (e.g., the onset of puberty)^48^ or to anatomical sex differences (e.g., the digit ratio between the second and fourth finger)^49,50^ have also been associated with autism. In addition, mothers with endocrine conditions that involve excess androgens, such as polycystic ovary syndrome (PCOS), are more likely to have an autistic child^51^. This finding has been replicated in four separate populations, in a meta-analysis, and is independent of other metabolic conditions such as obesity^52,53^. These findings provide further support for the prenatal sex steroid theory and indicate that the association between elevated sex steroids and autism likelihood can be attributed to an interaction between genetics, health conditions and the process of pregnancy.

The interaction of these factors may be particularly evident in the placenta. This regulates both maternal physiology and fetal development, producing growth factors, aromatising androgens and very high quantities of estrogens in all pregnancies. Interestingly, the placenta differs markedly according to the sex of the baby, with steroid sulfatase (a steroid that regulates the levels of DHEAS, which in turn, is converted into testosterone and estradiol) being lower on average in the placentas of boys, compared to girls^54^. Consistent with this, we found that the placental growth factor (PLGF) showed pronounced sex differences during pregnancy, and that male-like patterns in the levels of the PLGF are associated with higher autistic traits during childhood^55^. In addition, we found that sex differences in placental gene expression show a significant overlap with genes associated with autism on the X-chromosome^48^.

Placental complications, such as preeclampsia and pregnancy-induced hypertension are also more frequent in pregnancies of autistic children^56–58^, or of children with high autistic traits in later life^55^. Interestingly, the latter association may also interact with prenatal steroidogenesis, as placental complications were found to be more common in pregnancies with high testosterone in a subsequent study of an independent population^59^. Many of these placental complications often lead to premature birth, which has also been consistently associated with autism^56^, autistic traits^60^, as well as pregnancies of males in general^61^. Interestingly, the largest genome-wide-association-study (GWAS) of premature birth and gestational duration also showed significant genetic correlations between genetic variants associated with these traits, and the genotypes associated with high total testosterone levels and bioactive testosterone levels in women^62^.

Finally, exposure to endocrine-disrupting chemicals has also been associated with neurodevelopmental outcomes. These include statistical associations between autism and environmental pollutants or very fine pesticide-related particles^63,64^, as well as more complex compounds that disrupt steroid pathways^65,66^. However, the latter finding was not confirmed in an analysis that assessed exposures to multiple agents and the levels of these in maternal serum^67^. Given the wide prevalence of these agents in the environment and the recent discoveries that some can interact with placental function and steroidogenesis^68,69^, more research is warranted to replicate these findings in well-powered cohorts, using a variety of study designs (including sibling-controlled analyses to control for genetic confounding) before informing public policy.

Thus, elevated prenatal sex steroids could arise from a variety of sources during gestation and be attributed to multiple genetic and/or environmental factors that are also associated with autism. This elevation may functionally interact with both the sex of the fetus and with genetics, since autism is highly heritable and both rare and common genetic variants contribute significantly to autism likelihood^70^.

## Prenatal sex steroids and other phenotypes related to autism

Autistic people, on average, show a behavioural profile that can be described as fitting an extreme of the typical male profile, in relation to two key dimensions of psychological traits: cognitive empathy and systemizing. This is because typical girls on average develop empathy faster than boys^71,72^, typical boys on average score higher on tests of ‘systemizing’^72^, and autistic people on average score lower on empathy, and higher on systemizing^72,73^.

If this ‘male-type’ shift is mediated by high levels of prenatal sex steroids, then we would expect autistic people to show more ‘male-like’ profiles in other traits and developmental processes that show sex differences. In line with this, facial morphology has been found to be more ‘male-like’ in autistic children and in autistic adults, regardless of their sex assigned at birth^74,75^. A large neuroimaging study, using a machine-learning algorithm trained on typical sex differences, also found greater male-type shifts in the brain structure of autistic people, compared to non-autistic. Interestingly, this was associated with genetic patterns that involve genes regulated by sex steroid hormones such as testosterone and estrogens, as identified in other experimental studies^47^.

In addition, evidence from rodents and stem cells suggests that sex steroids may directly affect the development of the brain, since estrogens regulate aspects of neuronal proliferation and neurone outgrowth during development in humans^6,76^ and androgens increase neuronal proliferation in stem-cell-derived neural organoids^3^. The number of neurons in the brain differs between the sexes^77^, with men on average having 16% more neurons than women, and brains from autistic people have 67% more neurons than the brains of undiagnosed people^78,79^. The proliferative effect of androgens favours excitatory neuron subtypes and leads to an imbalance in the ratio between excitation/inhibition (E/I)^3^, which has also been associated with autism^80,81^. Interestingly, the target genes of androgens in developing human neurons are also enriched for autism-related genes^82^.

**Figure 5:**
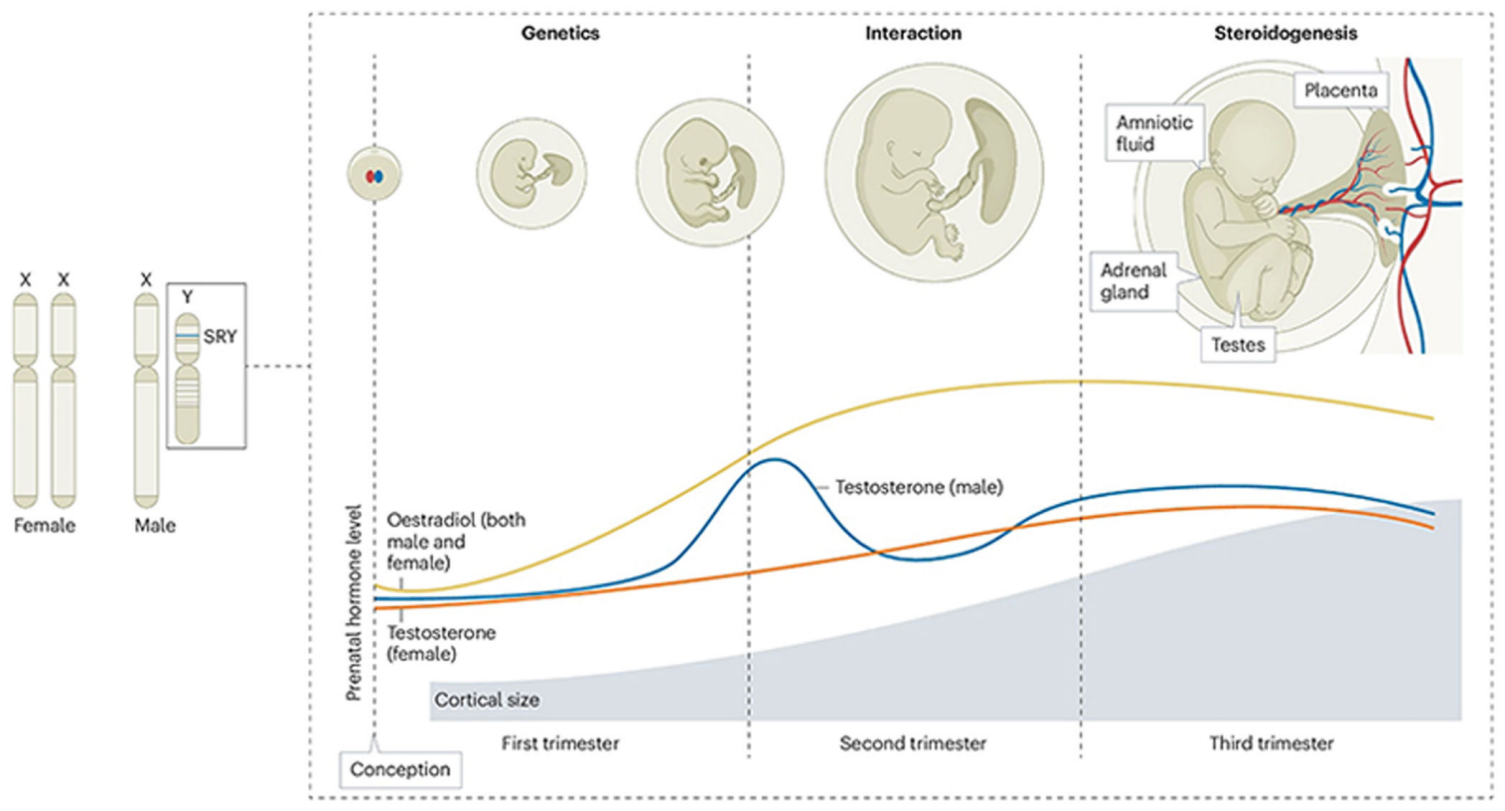
Prenatal interactions. Genetic and chromosomal factors interact with prenatal environmental to affect sexually dimorphic brain development. The coloured lines show approximate trajectories for steroid hormone levels, based on current clinical understanding^94,95^.

All these findings suggest that in humans, the interaction between sex steroid hormones and genetics is associated with changes to the rates of neuronal proliferation and apoptosis in the brain (Figure 5), and regulation of brain connectivity. This could potentially lead to imbalances between excitation and inhibition^83^, which may be particularly relevant in autism. This hypothesized prenatal hormonal pathway may thus provide a neuronal basis for the ‘extreme male brain’ theory of autism, which first studied psychological sex differences to understand the causes and prevalence of autism in both males and females.

## Implications for health

In a subset of autistic people, sex steroid levels may continue to show differences after birth^84^ and particularly around puberty^85^. Autistic women are more likely to report symptoms related to hyperandrogenaemia in adulthood, such as acne and history of hirsutism^86,87^ and to be diagnosed with hyperandrogenic conditions such as PCOS^51^. Endocrine pathology may then be an important feature of a subtype of autistic individuals, which could be contributing to their elevated risk of premature mortality^88^, warranting further research in terms of screening, early detection and treatments. However, any therapeutic option involving hormone regulation, is unlikely to be applicable during prenatal life, when steroid hormone levels are dynamic and highly sensitive to small variations, which can adversely impact the health of pregnancy or the normative sex differentiation of the fetus. For these reasons, research into the role of prenatal sex steroids in autism could contribute to postnatal screening, to tailored endocrine healthcare, but not to prenatal interventions of a preventative or therapeutic character, which our group also opposes on ethical grounds^89^.

## Conclusions

Several lines of evidence now support the prenatal sex steroid theory of autism. This is an important part of a complex cascade that includes genetic and post-natal social factors, and all may be associated with sex differences in autism likelihood. More research is needed to further examine evidence for causality and precise mechanisms, how prenatal sex steroids interact with genetics and if their elevation is linked to hormone-dependent conditions in later life, such as polycystic ovary syndrome, menstrual disorders or gastrointestinal pain, all of which are more common in autistic people^90^. In parallel, better support services are needed for all autistic people, irrespective of their biological sex or gender, to ensure that basic research can be translated to meaningful improvements in their health and well-being.

## Figures and Tables

**Figure 1:**
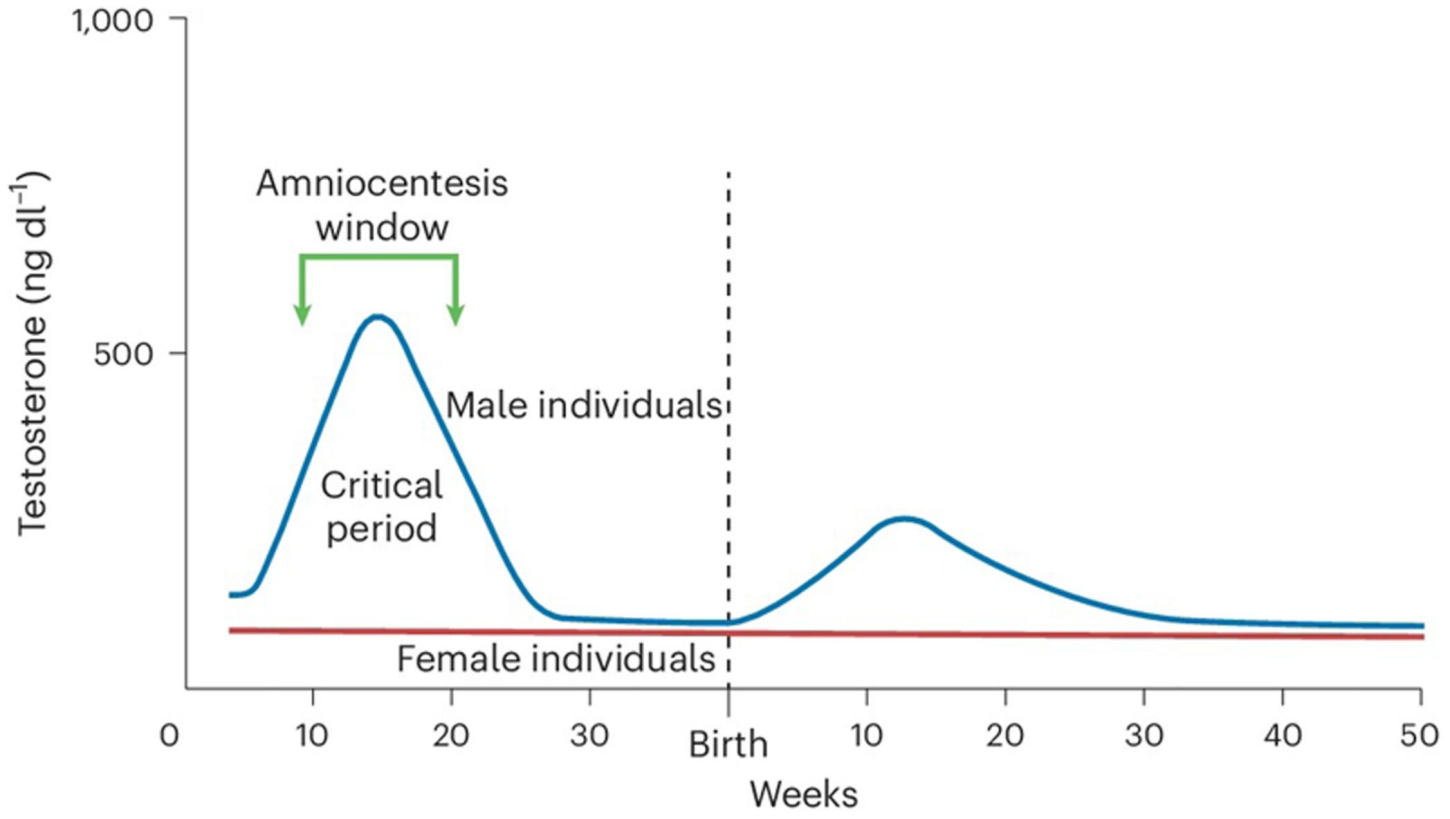
Testosterone surges during perinatal human development. The prenatal surge overlaps with the MWP (‘masculinization programming window’) and with when amniocentesis occurs. (Blue = males, orange = females). Figure adapted from Hines et al^91^.

**Figure 2:**
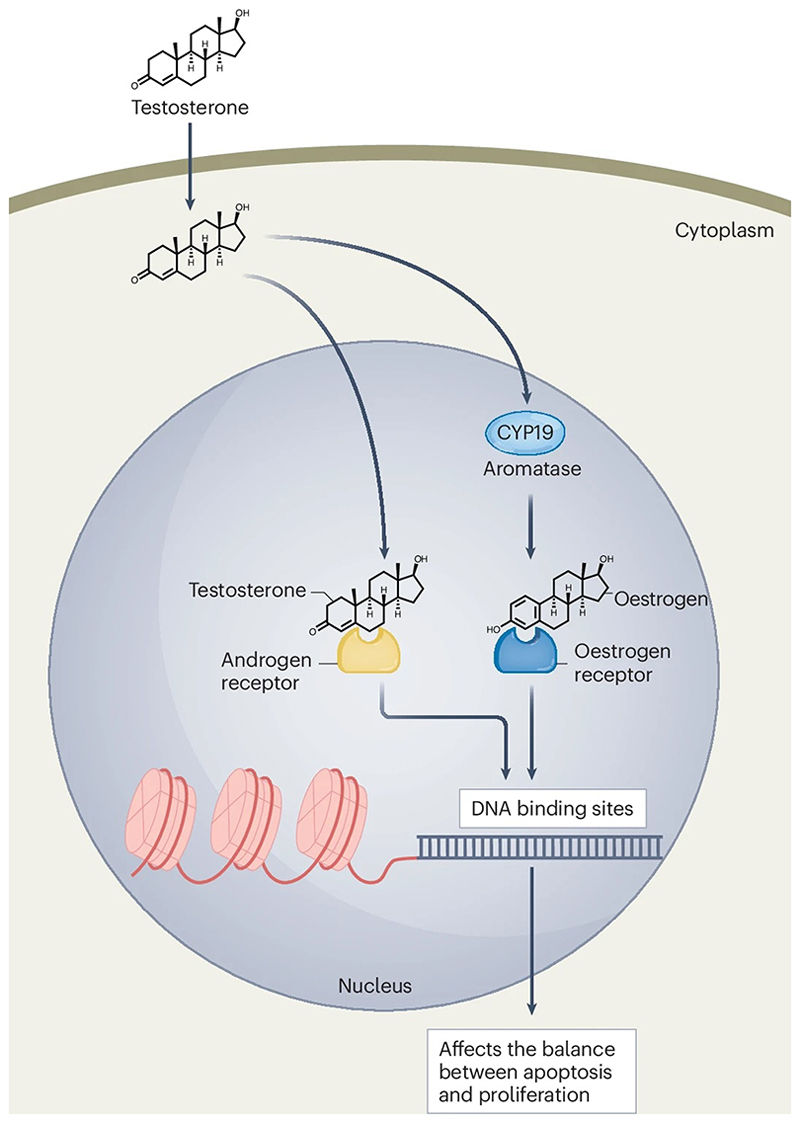
The molecular effects of testosterone. Testosterone enters the cell and exerts its effects directly via the androgen receptor or via aromatisation into estrogen via aromatase (CYP19) (outside or within the nucleus^92^) and by binding to an estrogen receptor. These receptors then can directly regulate gene expression in the nucleus and the rates of neuronal proliferation/apoptosis. Adapted from Morris et al (2004)^93^. Red circles represent histones.
